# Does Second Language Learning Promote Neuroplasticity in Aging? A Systematic Review of Cognitive and Neuroimaging Studies

**DOI:** 10.3389/fnagi.2021.706672

**Published:** 2021-11-12

**Authors:** Caitlin Ware, Sophie Dautricourt, Julie Gonneaud, Gael Chételat

**Affiliations:** ^1^PhIND Physiopathology and Imaging of Neurological Disorders, Institut Blood and Brain @ Caen-Normandie, Normandie Univ, UNICAEN, INSERM, U1237, Caen, France; ^2^CRPMS, Université de Paris, Paris, France; ^3^Neurology Department, University Hospital, Caen, France

**Keywords:** older adults, second language learning (L2 learning), executive functioning, neuroplasticity, cognitive reserve

## Abstract

As the population ages, understanding how to maintain older adults' cognitive abilities is essential. Bilingualism has been linked to higher cognitive reserve, better performance in executive control, changes in brain structure and function relative to monolinguals, and delay in dementia onset. Learning a second language thus seems a promising avenue for cognitive enhancement in older adults. Our review aims to determine whether learning a foreign language in later life improves cognition and promotes neuroplasticity. We screened articles from the Pubmed, Scopus, and Science Direct databases to identify interventional studies using second language training in senior participants, including either cognition or neuroimaging as outcome measures. A total of nine articles were found, with only one neuroimaging study. Results from these studies are inconsistent, but tend to suggest that second language learning is associated with improvement in attentional switching, inhibition, working memory, and increased functional connectivity. We discuss the implications of these results, and suggest new directions and methodological recommendations for future research.

## Introduction

With the population aging, incidence rates of dementia are on the rise. The World Health Organization's website predicts that by 2050, 152 million people could be living with dementia (World Health Organization, 2020). As effective drug treatments have yet to be developed, non-pharmacological interventions are currently the most viable option for preventing, or at least delaying, neurodegenerative diseases.

These interventions aim to boost or maintain cognitive and brain reserve (Stern, 2012). Overall, reserve refers to one's ability to better resist or cope with the accumulation of age-related or disease-related alterations. More specifically, the concept of cognitive reserve has been developed to account for interindividual variability in older adults' susceptibility to cognitive decline. For example, research has shown that some older adults are able to withstand greater brain atrophy before exhibiting cognitive deficits (Valenzuela and Sachdev, 2006). Higher reserve would support this greater resilience. Yet, it should be noted that reserve is a complex and multicomponent construct, and there is currently no consensus on its specific definition (Cabeza et al., 2018; Stern et al., 2020).

Nevertheless, research on the subject of reserve is abundant. Cognitive reserve and successful aging have been associated with lifestyle factors, including education (Stern, 2012), participation in leisure and social activities (Scarmeas and Stern, 2003), or physical exercise (Cheng, 2016). Of importance, cognitive reserve is not a static capacity, and is considered to be modifiable throughout life (Tucker and Stern, 2011).

Bilingualism, or the capacity to speak two languages fluently, has also been shown to contribute to cognitive reserve (Schweizer et al., 2012). Relevantly, dual-language use has been associated with executive functioning benefits, which have been observed in bilingual children, adults, and elders, even when the second language is acquired after childhood (Costa and Sebastián-Gallés, 2014). However, these cognitive advantages are debated, as some studies have failed to show differences in cognition between monolingual and bilingual adults (Paap et al., 2014; von Bastian et al., 2016; Nichols et al., 2020). Interestingly, differences may be more visible in older adults, as young adults already function at their peak (Bialystok et al., 2005). Thus, research focusing on bilingual older adults may shed more light on the cognitive benefits associated with bilingualism. Notably, it has been shown that lifelong use of more than one language could lead to enhanced cognition in later life (Bialystok et al., 2004). A few studies conducted in elderly individuals have evidenced an advantage in episodic memory, letter fluency (Ljungberg et al., 2013), semantic verbal fluency (Rosselli et al., 2000), as well as higher general intelligence (Bak et al., 2014a) in bilingual seniors. Moreover, many studies have revealed superior executive functioning in older bilinguals, notably in auditory attention tests (Bak et al., 2014b), cognitive inhibition, and task switching (Bialystok et al., 2006; Goral et al., 2015; Blumenfeld et al., 2016). These benefits are found primarily in tests like the Stroop, Flanker, and Simon tasks, in which colors, shapes, or arrows must be processed and selected, and others ignored or suppressed. In a meta-analysis including 28 articles (Armstrong et al., 2019), bilingualism was found to have a significant effect on seniors' cognitive inhibition. As cognitive inhibition, among other executive functions, is usually weakened with age (Hejazi et al., 2019), this finding suggests that speaking more than one language has a modulating effect on age-related cognitive decline in bilinguals.

Neuroimaging studies have also reported differences in brain measures between monolingual and bilingual older adults, showing higher gray matter volume (GMV) in the anterior temporal lobe and in the left inferior temporal gyrus in bilinguals, which correlated to second language naming ability (Abutalebi et al., 2014), as well as greater GMV in the anterior cingulate cortex (Abutalebi et al., 2015a; Del Maschio et al., 2018), and in the caudate nucleus, pre-frontal cortex, and inferior frontal cortex (Del Maschio et al., 2018). Higher GMV in the left and right inferior parietal lobule was also observed in older bilinguals compared to monolinguals, and this was correlated with naming ability and language exposure respectively (Abutalebi et al., 2015b). Using diffusion tensor imaging (DTI), greater axial diffusivity has also been detected in older bilinguals compared to monolinguals, specifically in the left superior longitudinal fasciculus (Anderson et al., 2018), along with greater fractional anisotropy in the corpus callosum, and the superior and inferior longitudinal fasciculus (Luk et al., 2011). Moreover, relative to older monolinguals, greater GMV and white matter volume in older bilinguals has been shown to correlate with more efficient executive functioning, as evidenced with superior inhibition and attentional performance, while no such correlation was found in monolingual groups (Olsen et al., 2015; Borsa et al., 2018).

Functional connectivity or activation differences between senior monolinguals and bilinguals have also been observed. With fMRI scans carried out during executive control tasks, greater functional connectivity in the frontoparietal control and default mode networks was found in older bilinguals relative to their monolingual peers (Grady et al., 2015). Moreover, older bilinguals switched faster than their monolingual peers during perceptual tasks, and fMRI imaging evidenced less activation in the left dorsolateral pre-frontal cortex, left ventrolateral pre-frontal cortex, and the anterior cingulate cortex, suggesting higher neural efficiency in bilingual seniors (Gold et al., 2013).

Altogether, cross-sectional studies suggest that bilingualism is likely to increase cognitive and brain reserve as it is associated with a beneficial effect on cognitive performance and cerebral integrity, notably in older adults. Despite the inherent discrepancies between lifelong dual-language use and later second language acquisition (SLA), bilingualism and SLA share important characteristics, notably considering the fact that learning a second language is a step toward bilingualism. Learning a second language at a later age may not lead to bilingualism, yet learning to use a foreign language could provide some cognitive benefits that may partly overlap with those associated with bilingualism. It has thus been hypothesized that SLA in older adults could be a promising avenue of cognitive training to promote healthy aging (Antoniou et al., 2013; Antoniou and Wright, 2017).

However, there is a common stereotype that SLA would be too challenging for older adults (Gómez, 2016). This perhaps stems from the widely cited critical period hypothesis, which postulates that there is a short window of time during childhood for successful language acquisition; but this hypothesis was developed in the context of first language acquisition, and does not necessarily apply to SLA (Singleton and Pfenninger, 2018). In fact, it has been shown that learning a new language, although more difficult after adolescence, is possible for older adults (Gómez, 2016; Kliesch et al., 2017; Hejazi et al., 2019). With age-related cognitive decline being gradual and varying greatly from person to person, more than a “critical” period, the notion of age-related changes affecting SLA could be better characterized as a “sensitive” period (Birdsong, 2018). Amongst the predictors of SLA ability in older adults, working memory capacity, over chronological age, has been shown to be a better predictor of second language success in seniors (Mackey and Sachs, 2012). What's more, implicit learning abilities, as opposed to those of explicit learning, have been shown to be more resistant to aging (Polony et al., 2016), and even improve with age (Ristin-Kaufmann and Gullberg, 2014), which could facilitate SLA in older adults.

Parting from the postulate that SLA in older adults is indeed possible, and based on previous evidence indicating a beneficial effect of bilingualism on older adults' reserve and resilience, this review aims to address the influence of late natural language learning on cognitive and brain aging. Artificial language interventions, although valuable in their own right, will not be included in this review as they do not share the same socio-cultural components as those of natural language. Moreover, although artificial and second language learning processes are related (Friederici et al., 2002), some research suggests that they depend on different mechanisms (Robinson, 2010), and therefore may affect the brain differently.

Moreover, learning a natural language has practical and social implications. Beyond the cognitive benefits, learning a foreign language in later life could be particularly enriching from a psychological perspective, as it could provide an outlet for socialization (Pfenninger and Singleton, 2019), opportunities for travel (Antoniou et al., 2013), intercultural communication (Kuklewicz and King, 2018), as well as a means of building self-esteem (Pot et al., 2018), and well-being (Matsumoto, 2019), which could in turn have positive effects on cognition (Allerhand et al., 2014).

While studies are scarce, some research has evaluated the cognitive and neuroanatomic effects of SLA through interventional designs in those over 60; yet results seem inconsistent. Therefore, it remains unclear whether learning a new language in late adulthood fosters cognitive efficiency and neuroplasticity. Our systematic review thus aims to clarify the effects of SLA on older adults' cognitive and cerebral functioning, by reviewing available evidence and analyzing the methodological quality of longitudinal studies with SLA interventions for seniors. We will present the results of our systematic review and discuss their implications in the context of both cognitive improvement or maintenance, and neuroplasticity associated with SLA. We will then outline some methodological shortcomings that should be taken into account for future studies, and finally address future directions based on ongoing studies or research protocols targeting the cognitive and neurological effects of language learning in seniors.

## Methods

### Search Strategy

Following the Preferred Reporting Items for Systematic Reviews and Meta-Analysis (PRISMA) model, we conducted the literature search using the following key word combinations in the Pubmed, Scopus and Science Direct databases up until November 12th, 2020: “language learning”/“second language”/“foreign language”/“second language learning”/“foreign language learning”/and “older adults”/“seniors”/“elderly”/“third age.” In each of the databases searched, the filter for article type was used, excluding review articles, book chapters, conference papers, and abstracts. For Science Direct and Scopus, when using the keyword “seniors,” “high school,” “college,” and “university,” the AND NOT command was used in order to avoid studies with younger adults. Four additional articles were identified through ResearchGate. See Table 1 for details.

**Table 1:** Search strategy.

**Search engine**	**Keywords**
Pubmed	(((“older adults” OR “elderly” OR “seniors” OR “third age”))) AND (((“language learning” OR “second language” OR “foreign language” OR “foreign language learning” OR “second language learning”)))
Science direct	(((“older adults” OR “elderly” OR “seniors” OR “third age”))) AND (((“language learning”))) AND NOT (((“high school” OR “university” OR “college”))) (((“older adults” OR “elderly” OR “seniors” OR “third age”))) AND (((“second language”))) AND NOT (((“high school” OR “university” OR “college”))) (((“older adults” OR “elderly” OR “seniors” OR “third age”))) AND (((“foreign language”))) AND NOT (((“high school” OR “university” OR “college”))) (((“older adults” OR “elderly” OR “seniors” OR “third age”))) AND (((“foreign language learning”))) AND NOT (((“high school” OR “university” OR “college”))) (((“older adults” OR “elderly” OR “seniors” OR “third age”))) AND (((“second language learning”))) AND NOT (((“high school” OR “university” OR “college”)))
Scopus	(((“older adults” OR “elderly” OR “seniors” OR “third age”))) AND (((“language learning”))) AND NOT (((“high school” OR “university” OR “college”))) (((“older adults” OR “elderly” OR “seniors” OR “third age”))) AND (((“second language”))) AND NOT (((“high school” OR “university” OR “college”))) (((“older adults” OR “elderly” OR “seniors” OR “third age”))) AND (((“foreign language”))) AND NOT (((“high school” OR “university” OR “college”))) (((“older adults” OR “elderly” OR “seniors” OR “third age”))) AND (((“foreign language learning”))) AND NOT (((“high school” OR “university” OR “college”))) (((“older adults” OR “elderly” OR “seniors” OR “third age”))) AND (((“second language learning”))) AND NOT (((“high school” OR “university” OR “college”)))

### Inclusion and Exclusion Criteria

**Figure 1:**
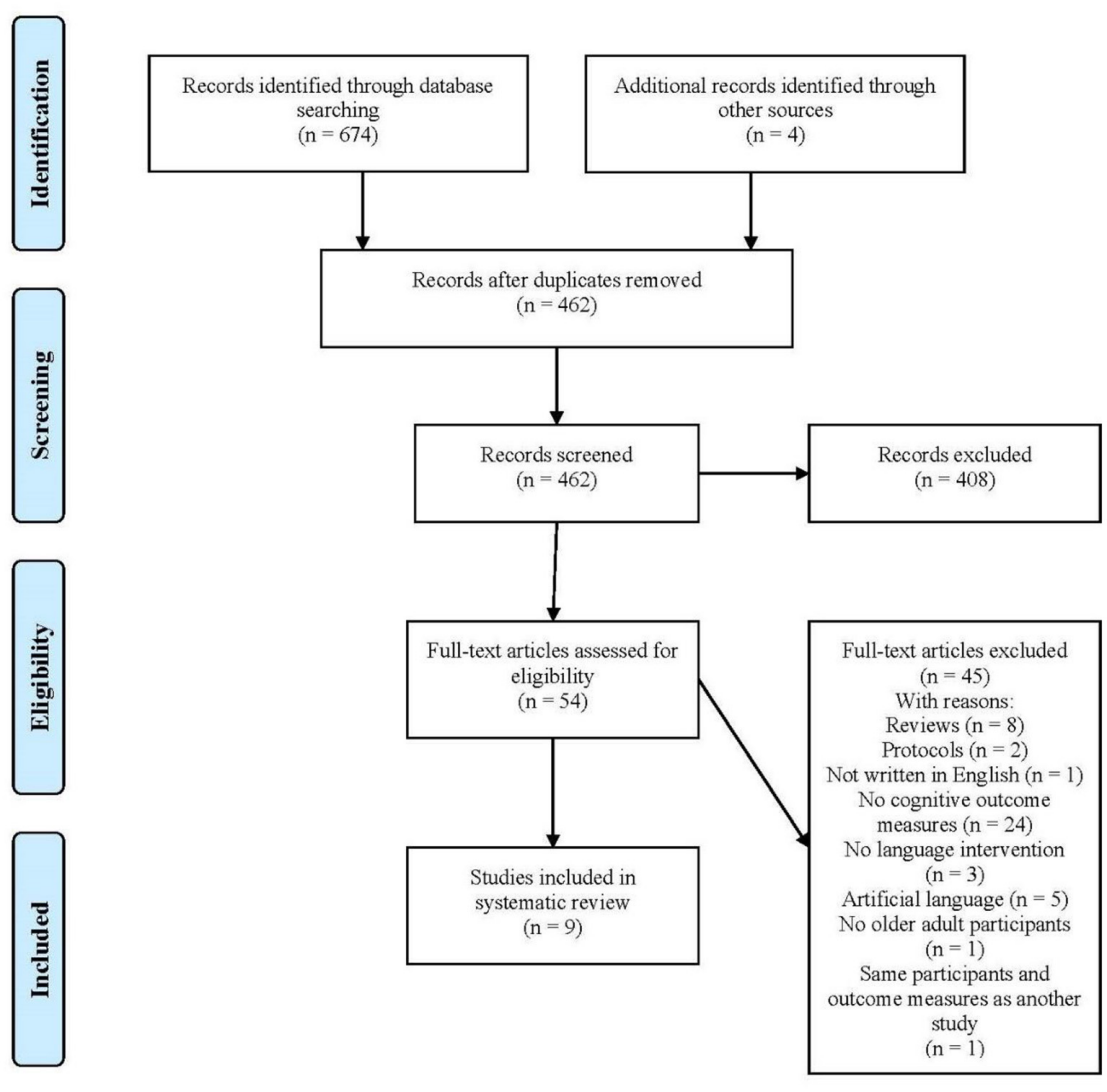
Prisma flowchart.

We selected studies based on the following criteria: (1) peer-reviewed original research; (2) including healthy participants over 60; (3) comprising a longitudinal second language intervention; (4) providing at least one outcome measure of cognitive functioning or neuroimaging; (5) published before the 12th of November, 2020. Studies with clinical populations, or those including only participants under 60, non-natural language interventions, exclusively qualitative data, written in a language other than English, as well as reviews, research protocols, abstracts, and preprints were excluded. See Figure 1 for details.

### Risk of Bias

In order to analyze the risk of bias (RoB) of the studies included in this review, we used the Study Quality Assessment Tools from the US Department of Health and Human Services (https://www.nhlbi.nih.gov/health-topics/study-quality-assessment-tools). Two independent raters (CW and SD) evaluated the studies with the *Quality Assessment Tool for Before-After (Pre-Post) Studies with No Control Group* or the *Quality Assessment of Controlled Intervention Studies*, depending on the study design. In case of inconsistencies between the two raters, results were further deliberated until agreeing upon a common score. RoB in the following part of this review will be discussed in terms of “methodological quality,” with high RoB corresponding to poor-quality studies and, reversely, low RoB corresponding to good quality studies.

For controlled intervention studies (randomized and non-randomized), there were 14 questions related to the study's randomization, blinding, baseline comparability of the experimental and control groups, drop-out and adherence to the assigned interventions, validation and pre-specification of outcome measures before analysis, sample size, and intention-to-treat analysis. A numerical scoring method was adopted, as previously determined by Cotelli et al. (2020), where the number of affirmative responses counted for one point and the sum of all responses was classified as follows: scores from 1 to 4 were rated as poor, 5–9 as fair, and 10–14 as good.

For interventional studies without a control group, there were 12 questions related to the clarity of the study's objectives, their selection criteria, the representability of their sample and its size, eligible persons' participation rate, the description and delivery of the intervention, the pre-specification and validation of outcome measures, assessors' blinding, drop-out percentage, pre-and post-statistical testing, and time-series design. The studies' RoB was scored using the same method, i.e., the sum of affirmative responses. The twelfth and final question of this scale concerned studies conducted on a group level such as a whole community or entire hospital patient population. Therefore, as this question did not apply to any of the included studies, we disregarded it, as did Cotelli et al. (2020). Thus, studies with scores from 1 to 4 were rated as poor, 5–8 as fair, and 9–11 as good. Full evaluations are provided in Supplementary Tables 1, 2.

## Results

### General Characteristics of Included Studies

After removing duplicates, we reviewed the pertinence of the 462 articles retrieved (Figure 1). After removing impertinent articles based on their titles, the abstracts and full texts of the remaining 54 articles were analyzed and studies were excluded if they were reviews (8), protocols (2), not written in English (1), as well as if they did not include cognitive outcome measures (24) or language interventions (3), only used artificial language training (5), did not include healthy older adults (1), or had the same participant sample and outcome cognitive measure of another included study (1). At the end of this selection process, only nine articles met our inclusion criteria.

Amongst the selected articles, eight included only cognitive outcome measures and one used both neuroimaging and cognitive outcome measures (Bubbico et al., 2019). A summary of the studies' participants and assessments is listed in Table 2, SLA and control interventions are detailed in Table 3, and the main outcomes and quality assessment scores are listed in Table 4.

**Table 2:** Summary of participants and assessments.

**Study**	**Country**	**Sample N**	**Groups (N and group)**	**Sex M/F**	**Age**	**Cognitive tests**	**SLA Test**
Bak et al. (2016)	Scotland	67	33 SL 16 active control 18 passive control	NR	18–78	TEA and its 3 subtests.	LBQ
Berggren et al. (2020)	Sweden	160	90 SL 70 control	60/100	65–75	Raven's matrices, the WASI-II, verbal intelligence, working and long-term associative memory tasks. *N*-back and numerical working memory test.	Vocabulary test
Bubbico et al. (2019)	Italy	26	14 SL 12 control	7/19	59–79	MMSE, VFT, TMTa and b, TMTab, the BMT, and FAB.	None
Klimova et al. (2020)	Czech Republic	42	20 SL 22 control	6/36	55–77	MoCA	Lex-tale
Long et al. (2019)	Scotland	105	NA	38/65	21–85	TEA and its 3 subtests.	LBQ
Pfenninger and Polz (2018)	Austria	12	NA	4/8	63–90	Stroop, A-K-T	C-test
Ramos et al. (2017)	Spain	43	26 SL 17 control	22/21	60–80	Switching paradigm test	Lex-tale
Ware et al. (2017)	France	14	NA	5/9	63–90	MoCA	None
Wong et al. (2019)	China	153	53 SL 51 games 49 music	23/130	60–85	ADAS-Cog, the auditory reading span, and the backward digit span tests, ANT, forward digit span, and Simon Task.	None

*SL, second language; NR, not reported; NA, non-applicable; TEA, Test of Everyday Attention; LBQ, Language Background Questionnaire; MoCA, Montreal Cognitive Assessment; ADAS-Cog, Alzheimer's Disease Assessment Scale96Cognitive Subscale; ANT, Attention Network Test; BMT, Babcock Memory Test; FAB, Frontal Assessment Battery; VFT, Verbal Fluency Test; A-K-T, Geriatric Concentration Test*.

**Table 3:** Detailed description of interventions.

**Study**	**Control group(s)**	**Description of SLA intervention**	**Duration, frequency, and total hours**
Bak et al. (2016)	Other intensive university courses Passive group	Summer intensive Gaelic course and additional evening entertainment (films, concerts, and conversation) at the National Center for Gaelic Language and Culture.	1 week, 14 h total.
Berggren et al. (2020)	Relaxation	Customized Italian course at an adult senior center, using a traditional text book (Olsson and Braconi, 2005) with grammar and verbal exercises, along with weekly vocabulary lists for memorization.	11 weeks, 2 weekly classes, 5 h/week, 55 h total.
Bubbico et al. (2019)	Passive	English language program with native teacher focusing on basic vocabulary skills and grammar, speaking and writing skills, including American and British culture, through classroom, homework, and team projects.	4 months, 1 90 min weekly class with 30 min homework, 32 h total.
Klimova et al. (2020)	Passive	Customized language course employing both traditional (vocabulary drilling) and non-traditional methods (problem solving and mind-mapping).	12 weeks, 3 weekly 45 min lessons, 27 h total.
Long et al. (2019)	NA	Summer intensive Gaelic course and additional and evening entertainment (films, concerts, and conversation) at the National Center for Gaelic Language and Culture.	1 week, 14 h total.
Pfenninger and Polz (2018)	NA	Intensive English course using the *Headway A1* (Soars and Soars, 2007) textbook, focusing on the understanding and use of everyday expressions, as well as vocabulary training.	4 weeks, 3 weekly 2-h sessions, 24 h total.
Ramos et al. (2017)	Passive	Basque at a Center for continuing education with native Basque-Spanish instructors, specialized in teaching adult learners.	8 months, 3 sessions per week, 176 h total.
Ware et al. (2017)	NA	Customized English class with media, and tablet exercises, focusing on oral comprehension and translation.	4 months, 2-h weekly sessions, 32 h total.
Wong et al. (2019)	Games (active) Music (passive)	English with *Rosetta Stone* Version 4, at a community center, along with bi-monthly group activities with English practice in a social setting.	6 months, 5 h per week, 120 h total.

**Table 4:** Summary of outcomes.

**Study**	**Study design**	**Time points measured**	**Outcome**	**RoB**
Bak et al. (2016)	CT	Baseline, a week later, and 9 months follow-up	Significant improvement in attentional switching (subtest 3) after just 1 week of SL training compared to passive controls, and maintenance of scores if Gaelic was practiced more than 5 h per week at follow-up 9 months later. All Gaelic learners showed significant improvement, regardless of age group.	7/14 Fair
Berggren et al. (2020)	RCT	Baseline and 11 weeks later	No significant differences were found between the SL group and the relaxation group, even though the SL group did demonstrate success in learning Italian vocabulary.	8/14 Fair
Bubbico et al. (2019)	RCT	Baseline and 4 months later	The MMS scores of the SL group remained stable, but the control group's scores significantly decreased. No other significant differences in cognition were found, although the SL group's functional connectivity increased in the right inferior and superior frontal gyrus, as well as in the left superior parietal lobule.	7/14 Fair
Klimova et al. (2020)	RCT	Baseline and 12 weeks later	Increases in MoCA scores were revealed in some of the participants in the SL group, yet they did not meet significance. Yet, decreases in scores in the SL group were also revealed, as well as increases in scores of the control group.	6/14 Fair
Long et al. (2019)	No CG	Baseline, and a week later	The TEA tests were found to be significantly correlated to Gaelic level. After the course, the beginner group evidenced the most improvement in TEA scores. Comparisons were made for different age groups, and all groups improved significantly in attentional switching and inhibition subtests 2 and 3.	5/11 Fair
Pfenninger and Polz (2018)	No CG	Baseline and 4 weeks later	Significantly improved Stroop and A-K-T scores for monolinguals, and significantly less errors on the language proficiency C-test. Qualitative results showed a positive effect on social life, improved memory, and boosted well-being.	8/11 Fair
Ramos et al. (2017)	CT	Baseline and 8 months later	No significant increases in switching ability among the intervention group were found in their test designed for the study.	3/14 Poor
Ware et al. (2017)	No CG	Baseline and 4 months later	No significant differences in MoCa and UCLA between pre-and post-intervention.	6/11 Fair
Wong et al. (2019)	RCT	Baseline and 6 months later and 3-month follow-up	Significant improvement was found for the active intervention groups, with the English group significantly improving in the ADAS-Cog, the auditory reading span, and the backward digit span tests. Yet the ANT, the forward digit span test, and the Simon task scores did not reach significance.	8/14 Fair

*For the quality assessment scores, it should be noted that the studies without control groups were scored over 11 points, while those with control groups were assessed with a 14-point scale. CT, Controlled trial; RCT, randomized controlled trial; CG, control group; SL, second language; MMS, Mini Mental State; TEA, Test of Everyday Attention; MoCA, Montreal Cognitive Assessment; ADAS-Cog, Alzheimer's Disease Assessment Scale -Cognitive Subscale; ANT, Attention Network Test; BMT, Babcock Memory Test; FAB, Frontal Assessment Battery; VFT, Verbal Fluency Test; A-K-T, Geriatric Concentration Test; UCLA, University of California Loneliness Assessment*.

The nine studies included in this review have a total of 622 participants, aged 18–90. Two studies included a large sample of adults 18 and over, including subgroups of older participants over 60 (Bak et al., 2016; Long et al., 2019), while the other studies only recruited older adults. Six studies had a control group, four of which were randomized. In these studies, the control groups included either passive controls that did not change their habits (Ramos et al., 2017; Bubbico et al., 2019; Berggren et al., 2020; Klimova et al., 2020) or active controls involved either in other university courses (Bak et al., 2016) or online games like Sudoku and crossword puzzles (Wong et al., 2019). Of note, these two last studies had both active and passive control groups, although in the former they were not assigned randomly, as participants chose their intervention group. The remaining three studies did not have control groups (Ware et al., 2017; Pfenninger and Polz, 2018; Long et al., 2019).

Six studies used English with Chinese, Czech, Austrian, French, and Italian participants, two used Gaelic with Scottish participants, one used Basque with Spanish participants, and another used Italian with Swedish participants. The teaching methods varied from online classes to traditional classroom settings (see Table 3 for details).

The interventions' duration was very heterogeneous, ranging from 1 week to 8 months, and the intensity of training programs varied from 14 to 176 h, with an average of ~55 h.

Only two studies used standardized second language proficiency tests before and after the intervention (Ramos et al., 2017; Pfenninger and Polz, 2018). One study used a vocabulary test, but only after the intervention (Berggren et al., 2020), while the other studies did not evaluate the second language proficiency attained at posttest (Bak et al., 2016; Ware et al., 2017; Bubbico et al., 2019; Long et al., 2019; Wong et al., 2019; Klimova et al., 2020).

All of the studies had at least one cognitive outcome measure; the long-term cognitive effects of language learning were evaluated in only two studies with follow-up measures after their second language interventions either 3 months (Wong et al., 2019), or 9 months later (Bak et al., 2016).

### Methodological Quality of the Studies

The nine studies fell into three different categories: randomized controlled intervention studies (*n* = 4), non-randomized controlled intervention studies (*n* = 3), and before-after (pre-post) intervention studies with no control group (*n* = 2); see the Methods section for details on the quality (RoB) assessment. Detailed evaluation can be consulted in Supplementary Tables 1, 2.

All studies scored as fair, except one that had a poor-quality score, or high RoB (Ramos et al., 2017). Although none of the studies had high enough scores to be considered good quality, based on the scoring guidelines of Cotelli et al. (2020), five studies scored higher than the average six points (scores ≥7), and we therefore refer to them here as the higher quality studies (Bak et al., 2016; Pfenninger and Polz, 2018; Bubbico et al., 2019; Wong et al., 2019; Berggren et al., 2020).

### Cognitive Outcomes of Language Learning

**Figure 2:**
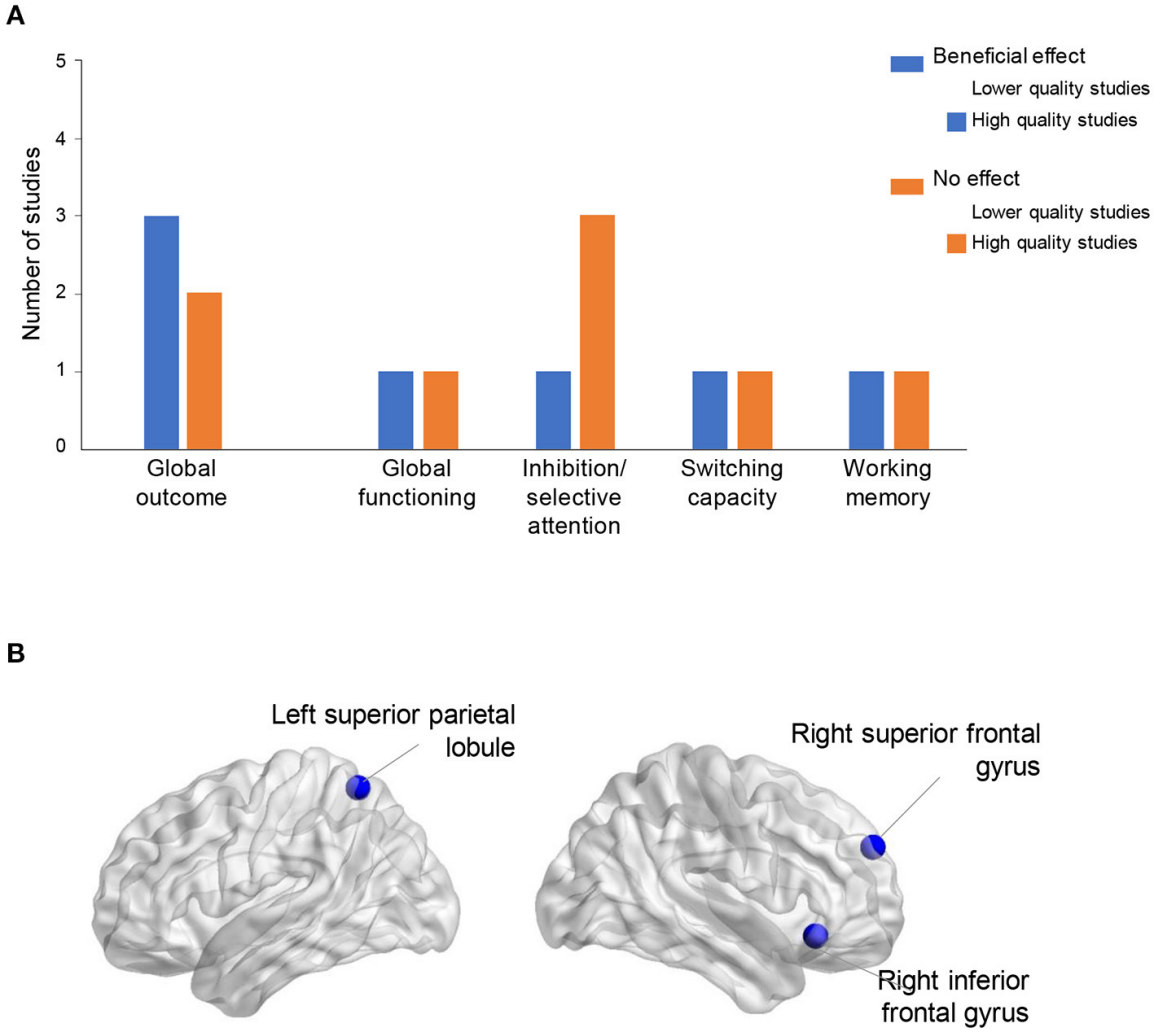
Effects of SLA on cognition and functional connectivity in older adults. **(A)** Combined results from the 9 reviewed studies on cognition. **(B)** Influence of second language learning on functional connectivity (Bubbico et al., 2019).

Focusing specifically on the five higher quality studies with executive functioning measures, mixed results have been shown for cognitive inhibition, attentional switching, and working memory (Figure 2A); with three of the studies evidencing improvement (Bak et al., 2016; Pfenninger and Polz, 2018; Wong et al., 2019), while the other two did not (Bubbico et al., 2019; Berggren et al., 2020). Yet, it should be noted that there are many methodological differences between these studies, with variation in intervention length and intensity, as well as diversity in terms of cognitive outcome tests. Longer (Wong et al., 2019), or more intensive interventions (Bak et al., 2016; Pfenninger and Polz, 2018) yielded positive results, while shorter, less intensive interventions did not (Bubbico et al., 2019; Berggren et al., 2020). Episodic memory (including associative, verbal and non-verbal episodic memory tests) was assessed in two higher quality studies and was not found to be significantly affected by second language interventions (Bubbico et al., 2019; Berggren et al., 2020). Additionally, verbal and spatial intelligence were not shown to improve either (Berggren et al., 2020). Yet, no other study in this review tested those specific intelligence and memory capacities (see Tables 2, 4). Finally, global functioning was found to improve after the second language intervention in one study using the ADAS-Cog (Wong et al., 2019). In another study using the MMSE, performances were preserved in the second language intervention group while they declined in the control group (Bubbico et al., 2019). In fact, the control and experimental groups' MMSE scores significantly differed at baseline, with the control group's scores significantly higher than those of the experimental group. However, with the decrease of the control group's scores and the maintenance of the intervention group's scores over time, there was no longer a significant group difference of scores at post-intervention.

When examining the lower quality studies (Figure 2A), one study showed significant improvement in cognitive scores, specifically in cognitive inhibition and attentional switching (Long et al., 2019), while the other three studies did not evidence cognitive improvement. In the lowest quality study, an original paradigm of switching, which was designed for the study, did not reveal changes in this cognitive function (Ramos et al., 2017). Additionally, some lower quality studies used brief tests of global functioning, like the MoCA (Ware et al., 2017; Klimova et al., 2020) and found no effect of the intervention. While these tests are validated and pertinent measures for patient populations, they may not be sensitive enough to capture subtle changes in cognition for healthy participants; these may be measured with more sensitive tests of global cognition such as the ADAS-Cog (see above).

### Language Learning and Functional Brain Connectivity

Only one study used neuroimaging outcome measures (Bubbico et al., 2019; Figure 2B). This higher quality study evidenced increased functional connectivity at post-intervention, both when compared to baseline connectivity and to controls, in the right inferior frontal gyrus (rIFG), the right superior frontal gyrus (rSFG), and the left superior parietal lobule (lSPL), while maintaining MMSE scores. Moreover, the increased functional connectivity in the rSFG was associated with increased global cognitive functioning scores, in the experimental group only.

## Discussion

### Summary of Main Results

Overall, our systematic review highlights mixed results regarding the cognitive effects of second language training in older adults (Figure 2A). As there are very few studies published on the subject to date, firm conclusions cannot yet be drawn. Nonetheless, four of the five studies with the highest quality scores (i.e., with the lowest RoB) did show significant increases in attentional switching (Bak et al., 2016), cognitive inhibition (Pfenninger and Polz, 2018), working memory (Wong et al., 2019), or functional connectivity (Bubbico et al., 2019), therefore providing some moderate evidence for increases in cognitive and cerebral functioning after short-term second language training in seniors. Nevertheless, in terms of methodological quality, none of the studies scored high enough to be considered good quality; therefore, the results should be interpreted with caution.

### Cognitive Effects of Second Language Learning in Seniors

When tallying the number of articles that show a positive effect of language learning interventions on cognition, the evidence for increases in scores of attentional switching and cognitive inhibition is the strongest, improvement in working memory and general cognitive functioning is moderate, while no indication of an effect on intelligence, verbal episodic memory or verbal fluency has been shown. These results can be interpreted in the context of bilingualism research as discussed in what follows.

Improvement in cognitive inhibition after second language learning is consistent with dual language processing. Cognitive control advantages associated with bilingualism are hypothesized to spring from having to juggle representations of two different languages in one's mind. The dual activation theory posits that while a representation of one language is activated, its correlate in the other language is activated at the same time. Thus, a bilingual would constantly have to inhibit one language while using the other (Green, 1998). Interference inhibition is thus essential for bilingual language use (Antoniou and Wright, 2017). This could explain the increases in cognitive inhibition scores in two studies (Pfenninger and Polz, 2018; Long et al., 2019), as learning a new language requires the inhibition of one's mother tongue (Long et al., 2019).

Likewise, the lifelong use of more than one language has also been shown to affect switching capacity (López Zunini et al., 2019), and this was also found to improve after language learning in two studies (Bak et al., 2016; Long et al., 2019). Yet, it could be argued that as the language learning interventions of these studies are quite short, practice effects could be responsible for the improvement at post-test. Nonetheless, after 9 months, the scores of attentional switching were maintained only for participants who practiced the second language more than 5 h per week (Bak et al., 2016), therefore suggesting that the results were not due to test-retest effects but to the amount of second language practice.

The lack of increases in verbal skills could also be interpreted in the context of bilingualism. Bilinguals have been shown to suffer from more tip-of-the-tongue states (Bialystok et al., 2008; Pyers et al., 2009), and to have smaller vocabularies in each of their tongues, as they simply have less time to master new vocabulary in each language (Gollan et al., 2009). Thus, verbal memory, verbal fluency, and verbal intelligence may not greatly increase through second language training.

Participants' previous language experience can influence the benefits of second language interventions, as suggested by the greater improvement of attentional switching in beginners compared to intermediate and advanced second language learners, evidenced in Long et al. (2019). This is in line with the “adaptive control hypothesis,” which posits that to adapt to more complex language contexts, control processes are required and these higher demands on the executive control system will lead, eventually, to improvement in executive functioning (Green and Abutalebi, 2013). Therefore, the pre-training language level, as well as the intensity of second language training, are important factors to consider.

Moreover, participants' language status, in terms of being mono-, bi-, or multi-lingual, is also of consequential significance, as those who speak more than one language may already benefit from the cognitive advantages associated with speaking multiple tongues. Learning a new language in later life may be most beneficial for those with no second language experience at all. This is consistent with the absence of increases in cognitive scores in studies with Swedish participants who demonstrated working knowledge of English (Berggren et al., 2020), or with bilingual participants (Pfenninger and Polz, 2018).

Overall, there is some variability in the effects of SLA interventions on cognition. However, studies with the highest quality tend to demonstrate a significant improvement in some executive functions.

Nonetheless, it should be noted that second language learning in older adults is not equivalent to lifelong bilingualism, and the cognitive advantages associated with later language learning may not be as pronounced in those who are only just starting to learn a new language than in those who have juggled different tongues from a much earlier age. Further research is needed to establish whether SLA in late life could actually contribute to cognitive reserve, as it has been suggested for lifelong bilingualism.

### Functional Brain Correlates of Second Language Learning

Whereas, behavioral measures do not always show significant improvement after SLA interventions, the only neuroimaging study conducted on older adults suggests that the effects of learning a new language can be detectable in the brain. After second language learning, connectivity has been shown to increase in the rIFG, rSFG, and lSPL (Bubbico et al., 2019). Interestingly, these regions have been shown to be involved in both the executive control and language networks (Tops and Boksem, 2011; Shomstein, 2012; Hu et al., 2016). Moreover, the connectivity increases were correlated with higher scores of global cognition (i.e., MMSE) in the intervention group, perhaps revealing cognitive maintenance. In fact, the maintenance of scores after the intervention, in comparison with passive controls, could be encouraging as learning may have a beneficial influence, not only through brain growth and improvements in cognitive performance, but also by reducing age-related brain alterations and cognitive decline (Nyberg et al., 2012).

These results align with previous studies in younger adults that suggest that language learning influences brain plasticity. For instance, language learning in younger adults has been shown to affect functional connectivity (Ghazi Saidi et al., 2013), GMV and cortical thickness (Legault et al., 2019). Promisingly, studies in young adults have evidenced increases in hippocampus volume after second language training (Mårtensson et al., 2012; Bellander et al., 2016). As the hippocampus plays an important role in episodic memory, and hippocampal atrophy is widely recognized as a biomarker of Alzheimer's disease (De Flores et al., 2015), increases in its volume, as a function of SLA, could be of significance in the face of age-related atrophy and cognitive decline. Furthermore, older bilinguals have been shown to have greater left hippocampal GMV than their monolingual counterparts (Li et al., 2017).

While these studies on SLA in younger adults seem encouraging, older adults might not rely on the same neural mechanisms when learning a new language, and therefore the same brain regions may not be affected in the same manner. For example, learning second language vocabulary, as in the memorization of foreign words, has been shown to affect the brain differentially as a function of age, with older adults evidencing greater activation in the left IFG, left lingual gyrus and cuneus, and younger adults showing greater activation in the left cingulate gyrus and the left caudate nucleus (Marcotte and Ansaldo, 2014). Relative to their younger counterparts, these different mechanisms may induce different patterns of brain plasticity in seniors.

### Limits

Altogether, although this review highlights some evidence for increases in executive functioning with language learning, it also points to inconsistencies, probably due to methodological differences across the different trials.

Some of the studies' methodological shortcomings should be emphasized: the lack of pertinent, sensitive, or validated cognitive measures, small sample sizes, lack of randomized active and passive control groups, absence of second language proficiency tests, as well as short or low intensity language training programs.

First of all, most of the lower quality studies scored below average due to small sample sizes or a lack of randomization. The inclusion of randomized active and passive control groups is very important for a study's credibility. This is particularly true when studying the elderly, who are more likely to decline over time. Indeed, the absence of modifications can either indicate an absence of a beneficial effect, or a maintenance of scores, which could be in fact a positive result; only the inclusion of a control group can help to unravel such results.

Secondly, it is of note that higher quality studies tended to use more than one cognitive outcome test and generally found a positive effect of language learning. In contrast, studies that were rated as lower quality used only one cognitive outcome measure, such as the MoCA, a brief test of global cognitive functioning, which greatly limited the possibility of capturing subtle cognitive changes. Another point is the choice of cognitive measures. Berggren et al.'s (2020) study did not show a significant effect on cognition, but they did not use tests that measure cognitive inhibition and switching, which have previously been shown to be affected by dual language use.

Thirdly, across the studies, a variety of tests were used to measure the same cognitive functions. Yet, tests that supposedly measure the same executive function, do not always yield similar results. For instance, the Simon, Stroop, and Flanker tasks, although sometimes used interchangeably, measure different subcomponents of attention, and therefore can produce different results (Dash et al., 2019). Only two of the studies in our review used the same executive functioning test (i.e., *TEA*), and both showed increases in attentional switching scores (Bak et al., 2016; Long et al., 2019). Most of the other studies used different tests, which complicates their direct comparison and may account for some of the discrepancies in results.

Fourthly, the inclusion of validated language proficiency measures is perhaps as equally important as the inclusion of appropriate cognitive measures; yet some of the studies had none at all. This is a major flaw in methodology if researchers aim to provide solid evidence of effects that are induced by actual language learning.

The differing intensities of the studies' language interventions could further account for some of the discrepancies in results. For instance, working memory improved in one higher quality study with a 6-month long intervention (Wong et al., 2019), and did not in another, also of higher quality, but proposing a shorter intervention of under 4 months (Berggren et al., 2020).

Finally, another point that merits consideration is how a foreign language is taught, given that social learning impacts the brain differently than traditional methods in SLA (Li and Jeong, 2020). For instance, it has been revealed in younger adults learning a foreign language that a larger impact on the brain is induced through social interaction than through media (Yusa et al., 2017).

Participants' language and cultural background is also of significance. Study protocols should include language background and demographic questionnaires, and recruit participants who share similar cultural and linguistic experiences, as the neurocognitive effects of learning might vary as a function of similarity to participants' mother tongues. The choice of target language is thus significant. In this respect, artificial language learning could also be of interest as it allows for the control of language similarity and exposure (Folia et al., 2010).

Further, motivation is a very important factor in SLA research (Ushioda, 2009), as it is strongly correlated with language learning success (Gardner and Lambert, 1972; Hernandez, 2006), as well as commitment to the interventions. Therefore, measures of motivation should be included in research protocols.

Ultimately, in terms of brain imaging measures, only one study to date investigated functional changes associated with SLA interventions, with resting-state functional MRI. Future studies should provide a greater overview on SLA functional substrates, but also on structural correlates of second language learning in older adults, by including pre-and post-intervention multimodal neuroimaging. While more costly than simple neuropsychological evaluations, only brain imaging will help to better understand the mechanisms by which SLA interventions can influence older adults' brain health.

### Future Directions and Recommendations

Based on the limitations of the studies reviewed, we recommend considering the following points in future research: Inclusion of randomized active and passive control groups, cognitive test batteries with sensitive evaluations that measure subtle changes in the executive functioning domains previously shown to be affected by second language use, language proficiency tests carried out at pre-and post-intervention, linguistic and demographic questionnaires, measures of motivation, and perhaps most importantly, pre-and post-intervention multimodal neuroimaging measures. Finally, teaching methodologies should be outlined carefully, with course duration and intensity justified within the context of previous SLA research. The influence of SLA interventions on cognition and brain health has been relatively neglected and the few existing studies provide inconsistent results, stressing the need for further investigation. The above methodological considerations should be taken into account in these future studies. Fortunately, three ongoing research protocols on the effects of second language training in seniors have been identified, and the quality of their methodologies appear to be superior to those included in our review.

A study currently being conducted in France entitled *Age-well*, part of the *Silver Santé Study European Project*, assesses healthy senior participants (>65 years old), randomly assigned to either a meditation, English training, or a passive control group for 18 months (Poisnel et al., 2018). This study, with its large sample size (137 healthy participants), is unprecedented as it is randomized with an active and passive control group, the former being an 18-month English intervention taught at the University of Caen, with a complete battery of cognitive tests, multimodal neuroimaging, psychological questionnaires evaluating emotions and quality of life, blood biomarkers, as well as sleep quality measures, including actigraphy and polysomnography. If we were to preliminarily calculate its RoB score, it would be rated as a high-quality study considering its randomization, inclusion of two active interventions (meditation vs. English training), and a passive control group, blinding of assessors, statistical power, and intention-to-treat analysis.

Another study, entitled *ENGAGE*, will examine the effects of a leisure-based Spanish or music intervention on 144 Canadian healthy seniors with memory complaints. Participants will be randomly assigned to an experimental condition involving cognitive training through either musical or language training, or an active control group involving low stimulating activities such as discussing documentaries and playing casual video games (Belleville et al., 2019). Their interventions will last 4 months, yet participants will be followed for 24 months and undergo episodic memory and attention tests, as well as psychological assessments, and structural and fMRI scans. In addition, cognitive reserve proxies such as apolipoprotein, brain-derived neurotrophic factor, catechol-O-methyltransferase, and scores on lifestyle questionnaires will be analyzed. Despite the absence of passive controls, a preliminary RoB rating for this study would also suggest a high quality given their outlined randomization and blinding procedures, as well as their large sample size.

Finally, another Canadian protocol called “*Boosting Cognitive Reserve Through Adult Second Language Acquisition with Duolin*go” is recruiting around 90 older adults for a 4-month randomized controlled trial in which participants will either (1) learn Spanish through Duolingo, (2) train with a computerized cognitive stimulation program, or (3) be part of a passive control group for 16 weeks. Their main outcome measures are tests of executive functioning, including the *n*-back and the Simon tasks. Among other secondary outcome measures, Spanish proficiency will be evaluated with the WebCAPE Online Spanish test before and after the Duolingo intervention. Although the protocol has not yet been published in a scientific journal, this study seems to be of high quality with the inclusion of an active and passive control group, fairly large sample size, and pertinent cognitive measures, as well as a second language proficiency test as an outcome measure.

## Conclusion

Although inquiry on the subject of SLA interventions in older adulthood is still in its infancy, there are some indications of cognitive benefits associated with foreign language learning in later life, especially for executive functions. Yet, the quality of the reviewed studies is fair at best, and research on the subject is sparse. The only neuroimaging study to date showed improvement in functional connectivity, yet without any other references, conclusions cannot yet be drawn. Further research with complete validated test batteries that include standardized language proficiency tests, randomization with passive and active control groups, and longer interventions with larger sample sizes should be carried out to expand upon these promising findings. The three identified ongoing clinical trials with the aforementioned methodological strengths should notably allow for significant advancements in the field.

## Data Availability Statement

The original contributions presented in the study are included in the article/Supplementary Material, further inquiries can be directed to the corresponding author/s.

## Author Contributions

CW, JG, and GC formulated the research questions. CW conducted the literature search. CW and SD assessed the quality and risk of bias of the included studies. All authors had an active role in drafting and revising the manuscript, and all authors approved the final draft for publication.

## Funding

This research was supported by the French Institute of Health and Medical Research (INSERM), Region Normandie.

## Conflict of Interest

The authors declare that the research was conducted in the absence of any commercial or financial relationships that could be construed as a potential conflict of interest.

## Publisher's Note

All claims expressed in this article are solely those of the authors and do not necessarily represent those of their affiliated organizations, or those of the publisher, the editors and the reviewers. Any product that may be evaluated in this article, or claim that may be made by its manufacturer, is not guaranteed or endorsed by the publisher.
