# Supplementary material for: Does Second Language Learning Promote Neuroplasticity in Aging? A Systematic Review of Cognitive and Neuroimaging Studies
Source: Front Aging Neurosci. 2021 Nov 12;13:706672. doi: 10.3389/fnagi.2021.706672 (PMC8633567; doi:10.3389/fnagi.2021.706672)
Supplement: Supplementary file 1 [file Table_1.docx]

**Supplementary material**

**Supplementary Table 1 Detailed RoB Scores for Controlled Interventional Studies**

| Author/Year | Bak et al. 2016 | Berggen et al. 2019 | Bubbico et al. 2019 | Klimova et al. 2020 | Ramos et al. 2017 | Wong et al. 2019 |
| --- | --- | --- | --- | --- | --- | --- |
| 1. Was the study described as randomized, a randomized trial, a randomized clinical trial, or an RCT? | No | Yes | Yes | Yes | No | Yes |
| 2. Was the method of randomization adequate (i.e., use of randomly generated assignment)? | No | NR | NR | NR | NA | Yes |
| 3. Was the treatment allocation concealed (so that assignments could not be predicted)? | No | NR | NR | NR | NA | Yes |
| 4. Were study participants and providers blinded to treatment group assignment? | No | No | No | No | No | No |
| 5. Were the people assessing the outcomes blinded to the participants' group assignments? | NR | NR | NR | No | NR | Yes |
| 6. Were the groups similar at baseline on important characteristics that could affect outcomes (e.g., demographics, risk factors, co-morbid conditions)? | Yes | Yes | No | No | Yes | Yes |
| 7. Was the overall drop-out rate from the study at endpoint 20% or lower of the number allocated to treatment? | Yes | Yes | Yes | Yes | NR | No |
| 8. Was the differential drop-out rate (between treatment groups) at endpoint 15 percentage points or lower? | Yes | Yes | Yes | Yes | NR | NR |
| 9. Was there high adherence to the intervention protocols for each treatment group? | Yes | Yes | Yes | NR | NR | Yes |
| 10. Were other interventions avoided or similar in the groups (e.g., similar background treatments)? | Yes | NR | Yes | NR | Yes | NR |
| 11. Were outcomes assessed using valid and reliable measures, implemented consistently across all study participants? | Yes | Yes | Yes | Yes | No | Yes |
| 12. Did the authors report that the sample size was sufficiently large to be able to detect a difference in the main outcome between groups with at least 80% power? | NR | Yes | No | No | NR | NR |
| 13. Were outcomes reported or subgroups analyzed prespecified (i.e., identified before analyses were conducted)? | Yes | Yes | Yes | Yes | Yes | Yes |
| 14. Were all randomized participants analyzed in the group to which they were originally assigned, i.e., did they use an intention-to-treat analysis? | No | No | No | Yes | NR | No |
| Final score | 7 | 8 | 7 | 6 | 3 | 8 |

Abbreviations: Not reported (NR)

**Supplementary Table 2 Detailed RoB Scores for Interventional Studies Without Control Groups**

| Authors/Year | Long et al. 2019 | Pfenninger & Polz 2018 | Ware et al. 2017 |
| --- | --- | --- | --- |
| 1. Was the study question or objective clearly stated? | Yes | Yes | Yes |
| 2. Were eligibility/selection criteria for the study population prespecified and clearly described? | Yes | Yes | Yes |
| 3. Were the participants in the study representative of those who would be eligible for the test/service/intervention in the general or clinical population of interest? | No | Yes | Yes |
| 4. Were all eligible participants that met the prespecified entry criteria enrolled? | Yes | No | NR |
| 5. Was the sample size sufficiently large to provide confidence in the findings? | NR | Yes | No |
| 6. Was the test/service/intervention clearly described and delivered consistently across the study population? | No | Yes | Yes |
| 7. Were the outcome measures prespecified, clearly defined, valid, reliable, and assessed consistently across all study participants? | Yes | Yes | Yes |
| 8. Were the people assessing the outcomes blinded to the participants' exposures/interventions? | NR | NR | NR |
| 9. Was the loss to follow-up after baseline 20% or less? Were those lost to follow-up accounted for in the analysis? | NR | Yes | No |
| 10. Did the statistical methods examine changes in outcome measures from before to after the intervention? Were statistical tests done that provided p values for the pre-to-post changes? | Yes | Yes | Yes |
| 11. Were outcome measures of interest taken multiple times before the intervention and multiple times after the intervention (i.e., did they use an interrupted time-series design)? | No | No | No |
| 12. If the intervention was conducted at a group level (e.g., a whole hospital, a community, etc.) did the statistical analysis take into account the use of individual-level data to determine effects at the group level? | NA | NA | NA |
| Score | 5 | 8 | 6 |

Abbreviations: Not reported (NR), not applicable (NA)
